# Items

**Published:** 1889-08

**Authors:** 


### ITEMS.

THE AMERICAN RHINOLOGICAL ASSOCIATION will hold its seventh annual meeting at Chicago, Ill., August 28th, 29th and 30th, 1889.

The committee on the examination of the inmates of insane asylums will make their report "on the relation of rinal inflammations to mind affections" at this session.

DR. R. S. KNODE, Sec'y, Omaha, Neb.

VOMITING IN PREGNANCY.—A writer in the *Lancet* says: I have not failed once for many years, by a single vesication over the fourth and fifth dorsal vertebræ, to put an end at once to the sickness of pregnancy for the whole remaining period of gestation, no matter at what stage I was consulted. The neuralgic toothache and puritus pudendi of the puerperal condition yielded as readily, and to one application.—*N. Y. Medical Times.*

ICE-WATER ENEMATA IN TREATMENT OF DIARRHŒA.—Dr. Robert M. Simon, writing in the *British Medical Journal*, states that this mode of treatment has frequently been adopted in cases of collapse occurring during diarrhœa in young children at the Birmingham General Hospital. He recommends that the ice should be dissolved in water and from two to three ounces injected. In his experience, which he says has been quite extensive, the immediate effect is good in producing sleep, as is important in the collapsed condition. Subsequently the effect upon the diarrhœa is also good, and it will rarely be found necessary to repeat the enema. It has sometimes been found expedient to give a few drops of brandy about the time of the injection, and perhaps internal medication should be continued. In his experience, no depression or bad effect has ever resulted.—*N. Y. Medical Times.*

DR. BROWN-SÉQUARD'S HYPODERMIC FLUID.—The extraordinary statements made by Professor Brown-Séquard as to the efficiency of hypodermic injections of fluid expressed from the testicles of young animals in senile debility have been to a certain extent confirmed by M. Variot, who made a communication to the Société de Biologie on June 29th. The patients chosen were debilitated men, aged 54, 56, and 68 years respectively, and they were not informed of the nature of the treatment adopted. In all three cases the injections were followed by general nervous excitement, increased muscular power, and stimulation and regulation of digestion. M. Brown-Séquard said that M. Variot's observations disposed of the objection that the results he had observed in himself were due to "suggestion."—*British Medical Journal.*

EXPERIMENTAL STUDIES ON HYPERPYREXIAS.—M. Ch. Richet, ("Jour. des soc. sci.," No. 20, 1888, has studied the influence of artificial hyperpyrexia, and the result of maintaining the high temperature for a considerable length of time. He says that animals whose temperature attains 45° C. [113° F.], and even 45·6° C. [114° F.], may survive if this thermic stage lasts for a short interval. But an animal succumbs if its temperature is about 43° C. [109·4° F.] for two hours ; a crisis occurs before death, the temperature gradually falls, the nervous system seems powerless to generate any more heat, and finally death ensues. Chloralization previous to the artificial hyperpyrexia was found by the author to hasten and assure death. The effect of chloral on the nervous system is considered as that of a depressing agent, which is added to that of the hyperpyrexia. This fact, it is stated, must be of importance in medicine, for the use of chloral seems dangerous in delirium, insomnia, and in other accidents occurring in hyperpyretic patients.—*N. Y. Medical Journal.*

CONTINENCE AND SYPHILIS.—*The Lancet*, commenting editorially upon our remarks regarding continence as a preventive of syphilis, adds: "Though Dr. Gowers' testimony to the importance of chastity as a means of health is the last great note sounded to Englishmen, it does not stand alone in medical literature. There is another voice which may be recalled here which will sound for generations yet, as characteristic in its ethical strength as in its medical and scientific authority. Sir James Paget, in his clinical lectures, speaking of patients that expect us to prescribe fornication, says: 'I would just as soon prescribe theft or lying, or anything else that God has forbidden. If men will practise fornication or uncleanness, it must be of their own choice and on their sole responsibility. . . . . . Chastity does no harm to mind or body; its discipline is excellent; marriage can be waited for; and among the many nervous and hypochondriacal patients who have talked to me about fornication, I have never heard one say he was better or happier after it; several have said they were worse; and many, having failed, have been made much worse.'"—*N. Y. Medical Record.*

A NEW SYMPTOM OF PERICARDITIS.—In some cases the diagnosis of effusion into the pericardium is difficult; and a symptom, first noticed by Bamberger, is said to be constantly present, and to aid materially in arriving at a correct conclusion. Puis, in the *Wiener medicinische Wochenschrift*, early in this year, has again attracted attention to the point. By percussion of the patient in sitting position, or when lying on the right side, there is a muffled tympanitic resonance or diminished resonance over the left side of the thorax behind, extending downwards from the angle of the scapula; and at the place of greatest loss of resonance there is distinct bronchial breathing and bronchophony, with increased vocal fremitus. If the patient is made to bend forward, a portion of the dullness completely disappears, another portion becomes tympanitic, and no bronchial breathing is heard. This change is more marked still if the patient assumes the knee-elbow position. The physical signs observed are ascribed to compression of the lower lobe of the left lung by the fluid in the pericardium, and is found chiefly in young adults with chests which are elongated or narrowed antero-posteriorly. The presence of pneumonia or pleuritis is contra-indicated by the alteration of the physical signs when the position of the patient is changed.—*British Medical Journal.*

THE INFLUENCE OF THE PNEUMOGASTRIC NERVES ON THE URINARY SECRETION.—M. M. Arthaud and Butte ("Jour. des soc. sci.," No. 20, 1888) conclude from their recent experiments that the pneumogastric nerves influence the urinary and, to a certain degree, the other visceral secretions. An excitation of the peripheral end of the vagus caused a diminution of the biliary secretion; the deep vessels of the stomach contracted after an excitation of the same nerve. An excitation of the peripheral end of the cervical pneumogastric, after excision of the branches proceeding from the inferior cervical ganglion, caused a noticeable variation of the cardiac pressure, though the rhythm remained unchanged. The urinary secretion of one kidney is not equally influenced by an excitation of the right and left vagus. An excitation of the peripheral end of the right vagus, somewhat above the diaphragm, gave for a definite length of time in the right kidney zero, and in the left 12 c. c. of urine; the same stimulus on the left side gave 11 c. c. from the right, and 8 c. c. from the left kidney. The conditions were not influenced by whether the stimulus was applied to the vagus above or below the heart. The facts lead the authors to think (1) that the variations of the cardiac rhythm do not influence the phenomena of secretion, and that this is an indirect demonstration of the special vasomotor action of the pneumogastric on the kidneys; (2) that a crossed distribution of the vagi exists.—*N. Y. Medical Journal.*

ACTION OF COCAINE ON THE BODY TEMPERATURE.—As interesting as those chemical bodies which reduce temperature in the febrile state are those which when introduced into the bodies of animals or of man produce fever. It is possible that the investigation of the action of these fever-producers may lead to some clearer ideas than we have at present on the influence of the central nervous system on heat-production and heat-loss. According to Ott and Colmar, peptones and albumoses, when introduced in sufficient quantity into the circulation of the blood, cause a rise of body temperature by affecting the cerebral heat centers. We may therefore have a "peptone fever." Mosso, in an elaborate paper on cocaine published last year, showed that cocaine also increased the body temperature; and he considered it the most energetic body yet known that possesses this action. Curiously enough, he found that, although chloral hydrate antagonized many of the physiological effects of cocaine, it did not counteract the febrile action, a result all the more astonishing, since one of the most marked effects of chloral is to reduce the body temperature. Reichert, in a recent communication to the *Universal Medical Magazine*, has further experimented with cocaine. He found that in dogs a dose of 0.0025 gramme of hydrochlorate of cocaine per kilo of body weight raises the temperature 0.2° to 0.5° C., while a larger dose, 0.02 gramme per kilo, causes a rise of 4° C. There is, however, no direct relation between the dose of cocaine and the effect on the body temperature; small doses may have a greater effect than large, and *vice versâ*. With large non-fatal doses, however, the temperature may remain high for as long as eight hours; so that we have not merely an evanescent rise of the temperature, but the development of an actual febrile state. As in the case of peptone, section of the medulla from the spinal cord prevents this action of cocaine. The effect is therefore on the heat centers in the brain. Calorimetric investigation showed that there was increased heat production at first, followed by increased loss of heat. If the animal were curarised, however, it was found that instead of the increase of production there was an increase of loss of heat; so that curare in this respect seems to antagonize the effect of cocaine.—*British Medical Journal.*

BROWN-SEQUARD'S NEW REJUVENATOR.—Brown-Séquard's statements regarding the stimulating power of testicular juice, which were received with much incredulity, have been in a measure confirmed through further experiments by M. Variot, who made a communication to the Société de Biologie on June 29th. The patients chosen were debilitated men, aged fifty-four, fifty-six and sixty-eight years respectively, and they were not informed of the nature of the treatment adopted. In all three cases the injections were followed by general nervous excitement, increased muscular power, and stimulation and regulation of digestion.

Brown-Séquard is not the first to lay stress on the potency of juices artificially extracted from the generative organs. Horace, says *The Medical Press*, in one of his odes, beseeches the witch Canidia to reveal to him the secret of the draught which she prepared at night by crushing in a mortar pieces of flesh torn from the most fiery horses of Rome, and the patricians, says the Latin poet, used this mysterious liquid with great confidence. Consequently M. Séquard is but an humble successor of Canidia ! It would not be at all surprising if the testicles contained some stimulating leucomaine. We know that the salivary glands can generate a poison, and Dr. Wooldridge found an active principle in the thymus gland which caused a rapid clotting of the blood when injected into the veins. The toxic agents in the urine secreted at night are said to be stimulating and anti-hypnotic. There is not so much known about the leucomaines, and possibly Brown-Séquard has hit upon one of them. That his discovery, if it is one, is more than the discovery of some stimulating product of tissue change, we do not believe. But it will no doubt give a great impulse to the consumption of lamb fries.—*N. Y. Medical Record*

DRY CHLORIDE OF SODIUM IN THE TREATMENT OF SUBINVOLUTION OF THE UTERUS.—In those cases of subinvolution of the uterus where for any reason operative measures are withdrawn advisably, Dr. Hal. C. Wyman has found the treatment by dry applications of sodium chloride to the swollen cervix most satisfactory. The formula he uses is this:

℞ Chloride of sodium.................... ℥ j.
Powd. slippery elm bark............. ℥ iij.
Powd. hyoscyamus leaves............ ʒ j.

Mix and rub in a hot and dry mortar until thoroughly desiccated.

This is applied to a diseased cervix uteri in quantities equal to an ordinary teaspoonful once every other day, and sometimes oftener.—*N. Y. Medical Times.*

VERMONT MICROSCOPICAL ASSOCIATION,
BURLINGTON, VT., June 21, 1889.

*Messrs. Editors*:

GENTLEMEN:—The tremendous strides which Microscopial Science has taken the past few years, has resulted in discoveries of the greatest possible good to the public. The truth of the germ theory—that disease and death are caused by micro-organisms—is dependent wholly upon microscopic investigation, and the best minds in the land are constantly working upon this great subject.

To encourage these workers and stimulate new discoveries, we announce the offer of a prize of $250 for each discovery of a new disease germ.

As there are, undoubtedly, many quiet, earnest and able workers in this field of science that we cannot reach with this announcement, but who may see the columns of your paper, we trust you will insert the enclosed, or some similar notice. Any facts, figures or information upon this subject, which you, as journalists, may at any time desire, we shall be pleased to furnish.

Yours truly,

C. SMITH BOYNTON, A. M., M. D.,
Secretary.

CHANGES IN FACULTY OF COLLEGE OF PHYSICIANS AND SURGEONS, BALTIMORE, M. D.—The Faculty of the College of Physicians and Surgeons of Baltimore held a meeting last week to fill the vacancies created by the deaths of Professors John S. Lynch and Oscar J. Coskery and the retirement of Professor A. B. Arnold, who has removed to San Francisco. Prof. Thos. S. Latimer was transferred to the chair of principles and practice of medicine and clinical medicine; Prof. Chas. F. Bevan to the chair of principles and practice of surgery and clinical surgery; Prof. J. W. Chambers to the chair of operative and clinical surgery, and Prof. George H. Rohé to the chair of obstetrics and hygiene. Prof. Thos. Opie will continue as professor of diseases of women and dean of the faculty. To fill vacancies created by these transfers new professors were elected as follows: Prof. Henry Sewall, of the University of Michigan, to the professorship of physiology; Dr. George J. Preston to the professorship of anatomy, with the diseases of the nervous system, as a clinical branch of instruction. Dr. N. G. Keirle was elected as lecturer on legal medicine, in addition to his demonstrations in pathology; Dr. George Thomas as lecturer on diseases of the throat and chest; Dr. G. A. Liebig, Jr., of Johns-Hopkins University, lecturer on medical electricity, and Dr. J. H. Branham, demonstrator of anatomy. Drs. L. F. Ankrim, Frank C. Bressler and F. G. Moyer were appointed assistant demonstrators, and Dr. R. G. Davis, prosector of anatomy. Prof. Sewall, who comes here from the University of Michigan, is an old Baltimorean, and was for several years demonstrator of biology in Johns-Hopkins University. All the other appointees are residents of this city. As an evidence of esteem on the part of his colleagues, Prof. Arnold was elected emeritus professor of clinical medicine on his retirement.

EXPLANATION.—The following explains itself.—J. S. Todd, M. D.:

*Dr. J. S. Todd, Dear Sir:* You can never know how sorry I am that my ignorance has placed you in a bad light before the regular profession. As you know, I am a student of medicine, trying to make an honest living and enough to pay as I go. Your testimomial to the truss, which I am the agent for was a private communication and should not have been incorporated in my pamphlet without your consent, which was not obtained. But to show you that I meant no harm, you received from me one of the first and only 200 copies that were circulated. I have at your request, destroyed all the other copies and will send out no more.

There are certain grave errors in the testimonial as published; also prominent among them, is the word, "treatment." You said you approved of my appliance—not treatment. Again, you did not sign your name as President of the Georgia Medical Society, (as it is printed), or association, or in any official capacity whatever.

The mistakes in testimonials were due mostly to carelessness on the part of the printer.

C. E. MCCANDLISS.

